# Development and performance verification of a 3-D position-sensitive Compton camera for imaging MeV gamma rays

**DOI:** 10.1038/s41598-019-54862-z

**Published:** 2019-12-06

**Authors:** Hiroki Hosokoshi, Jun Kataoka, Saku Mochizuki, Masaki Yoneyama, Soichiro Ito, Hiroaki Kiji, Fumiya Nishi, Shuji Miyamoto, Tatsushi Shima

**Affiliations:** 1 Waseda University, Graduate School of Advanced Science and Engineering, Tokyo, Japan; 2 University of Hyogo, Laboratory of Advanced Science and Technology for Industry, Hyogo, Japan; 3 Osaka University, Research Center for Nuclear Physics, Osaka, Japan

**Keywords:** Astronomy and planetary science, Astronomy and astrophysics, Astronomical instrumentation

## Abstract

In gamma-ray astronomy, the 1–10 MeV range is one of the most challenging energy bands to observe owing to low photon signals and a considerable amount of background contamination. This energy band, however, comprises a substantial number of nuclear gamma-ray lines that may hold the key to understanding the nucleosynthesis at the core of stars, spatial distribution of cosmic rays, and interstellar medium. Although several studies have attempted to improve observation of this energy window, development of a detector for astronomy has not progressed since NASA launched the Compton Gamma Ray Observatory (CGRO) in 1991. In this work, we first developed a prototype 3-D position-sensitive Compton camera (3D-PSCC), and then conducted a performance verification at NewSUBARU, Hyogo in Japan. To mimic the situation of astronomical observation, we used a MeV gamma-ray beam produced by laser inverse Compton scattering. As a result, we obtained sharp peak images of incident gamma rays irradiating from incident angles of 0° and 20°. The angular resolution of the prototype 3D-PSCC was measured by the Angular Resolution Measure and estimated to be 3.4° ± 0.1° (full width at half maximum (FWHM)) at 1.7 MeV and 4.0° ± 0.5° (FWHM) at 3.9 MeV. Subsequently, we conceived a new geometry of the 3D-PSCC optimized for future astronomical observations, assuming a 50-kg class small satellite mission. The SΩ of the 3D-PSCC is 11 cm^2^sr, anticipated at 1 MeV, which is small but provides an interesting possibility to observe bright gamma-ray sources owing to the high intrinsic efficiency and large field of view (FoV).

## Introduction

Technologies for imaging gamma rays in astronomy have been developed in both the high and low energy ranges. For example, Imager on-Board the INTEGRAL (International Gamma Ray Astrophysics Laboratory) Satellite (IBIS), launched in 2002, demonstrates good sensitivity in soft gamma rays up to sub-mega-electron-volt energy levels using a coded aperture mask^1^. INTEGRAL-IBIS provided a sky map between 17 and 1,000 keV with 12’ (FWHM) angular resolution and has revealed several soft gamma ray sources so far^2^. Moreover, the Large Area Telescope (LAT) aboard the Fermi Gamma-ray Space Telescope was launched in 2008 for imaging high-energy gamma rays between 100 MeV and 300 GeV^3^. The main detectors of Fermi-LAT were silicon strip detectors (SSDs) to measure the tracks of electrons and positrons. Interaction with photons in this energy range is mostly pair production, and therefore the tracking detectors possess high sensitivity for gamma rays at more than 100 MeV. Fermi-LAT achieved angular resolution of ~5° at 100 MeV and ~0°.8 at 1 GeV, which led to the discovery of many unidentified sources^4^. Between these energy ranges, however, 1–10 MeV gamma rays can easily penetrate a collimator such as a coded aperture mask. Therefore, it is impossible to acquire images using photoabsorption within the detector. In addition, pair production does not occur adequately, and therefore, it is difficult to obtain a back-projection image using tracking detectors such as SSDs. A Compton camera is commonly used for identifying the direction of MeV gamma-ray sources based on Compton kinematics. The Compton Telescope (COMPTEL), which was a large Compton camera loaded on the Compton Gamma Ray Observatory (CGRO), demonstrated the highest sensitivity in the 1–10 MeV range^5^. However, the sensitivity of the MeV-energy range is currently a few orders lower than that of other energy ranges. CGRO-COMPTEL succeeded in producing a 1–30 MeV image of the entire sky for the first time; however, only 32 gamma-ray sources have been detected from this so far^6^.

Establishing a method to image 1–10 MeV gamma rays is of great significance because there is a substantial number of nuclear gamma-ray lines in this narrow energy range. In astrophysics, radioactive isotopes that are synthesized at the core of stars emit nuclear gamma rays when they decay. This band of nuclear gamma rays has even been detected in actual observation. For example, a diffused image of 1.807 MeV gamma rays from ^26^Al was obtained by both CGRO-COMPTEL and INTEGRAL-SPI^7,8^. The 1.807 MeV image appears concentrated on the galactic plane, which indicates nucleosynthesis is still taking place in our galaxy. In addition, 1.156 MeV gamma rays from ^44^Ti were detected from a region of young supernova remnants, Cassiopeia A^9^. Nuclear gamma rays are also emitted via nuclear excitation and de-excitation when cosmic protons interact with the interstellar medium^10^. Therefore, even spatial distribution of cosmic rays and interstellar medium can be revealed by detecting these nuclear gamma rays. Many de-excitation lines are expected to come from the galactic center region^11^. At present, however, these have neither been detected nor confirmed.

A Compton camera usually consists of a scatterer and an absorber where an incident photon undergoes Compton scattering and photoabsorption, respectively. The direction of a source is calculated using the energy deposit of the scatterer and absorber, and its position based on Compton kinematics. Although there are various choices of detectors as the scatterer and absorber^12–15^, our group adopted Ce-doped Ge_3_Al_2_Ga_3_O_12_ (Ce:GAGG) scintillators for environmental survey and medical use^16,17^. The properties of Ce:GAGG scintillators, such as radiation tolerance, have already investigated in a study that confirmed the suitability of Ce:GAGG for use in space satellite missions^18^. The high density of Ce:GAGG, combined with a compact configuration of the camera, can realize a high intrinsic efficiency, but in general, a scatterer has to be set considerably apart from an absorber to achieve a high angular resolution. In fact, in the case of CGRO-COMPTEL, the scatterer and absorber were placed 1.5 m apart from each other. The large structure also enabled the measurement of Time of Flight (ToF) to prevent the order of interactions from being misidentified. In contrast, our group developed a 3-D position-sensitive scintillator which can determine the 3-D position of gamma-ray interaction with an accuracy of ~1 mm^19^. Owing to this configuration, we can achieve high angular resolution even when the scatterer and absorber are placed as close as possible to each other. Although ToF cannot be measured in the compact configuration, back-scattering gamma rays from the absorber are avoided using the energy cut of the scatterer. Various methods to reduce background contamination have been considered, and we successfully proved these methods could exclude the background events and improve the quality of the MeV gamma-ray images^20,21^.

In this study, we used the prototype 3D-PSCC that was originally developed for MeV gamma-ray imaging in proton therapy^21^. To verify the performance of the prototype 3D-PSCC, we irradiated the camera with a MeV gamma-ray beam to mimic signals from celestial gamma-ray sources. Then, a clearly separated image of gamma rays from each incident angle, 0° and 20°, was obtained in the experiment. Subsequently, we considered the optimization and upsizing of the prototype 3D-PSCC based on the Geant4 simulation, assuming a payload of 50-kg class small satellite for future astronomical observations. Although the detection area is still small, we show that even a small satellite mission may provide a unique opportunity to open new energy windows toward MeV gamma-ray astronomy.

## Results

### Imaging of 1.7 and 3.9 MeV gamma-ray beams

**Figure 1:**
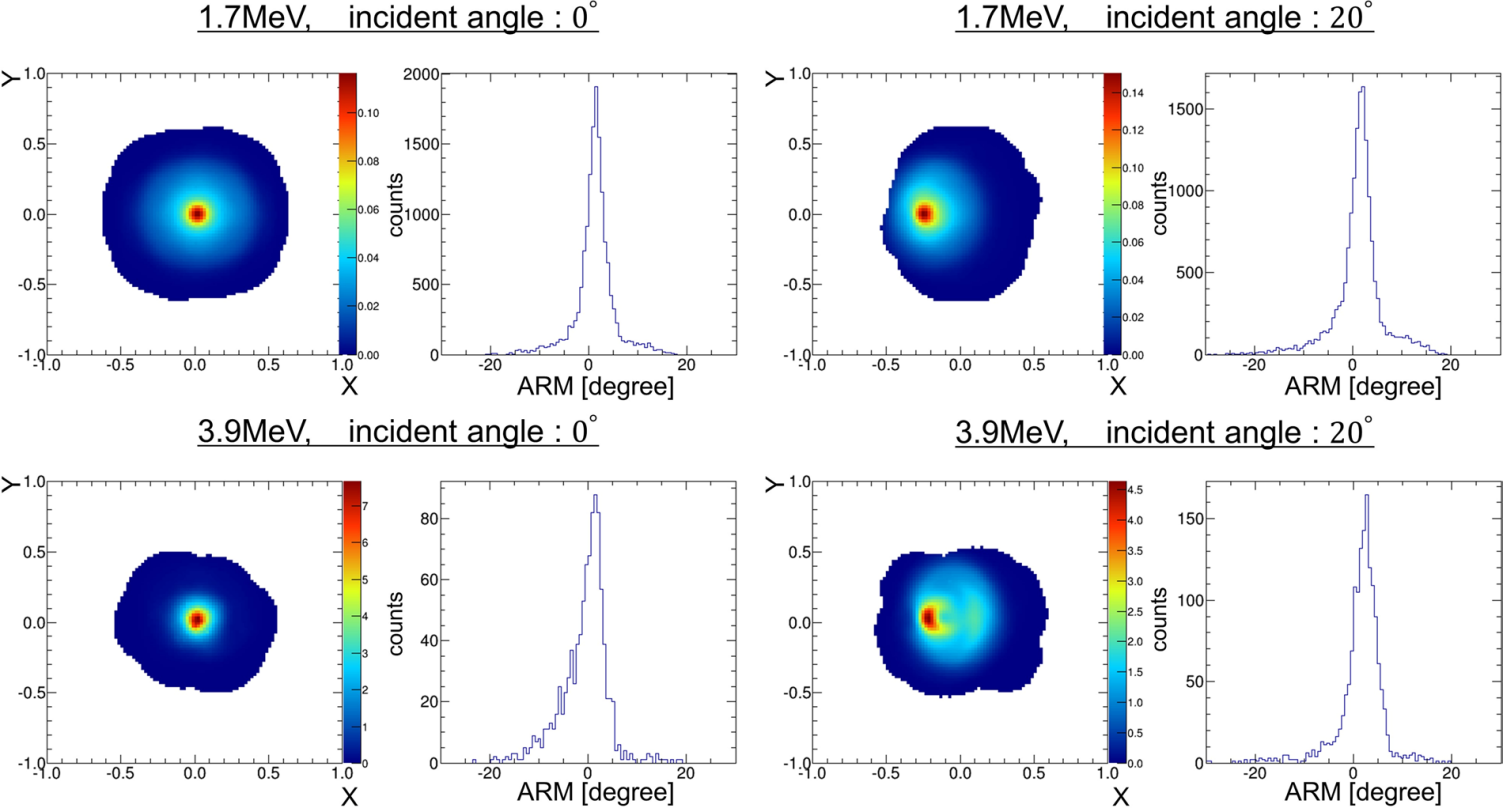
Experimental images and ARM spectra of the 1.7 MeV (*upper*) and 3.9 MeV (*lower*) gamma-ray beams irradiating from an incident angle of 0° (*left*) and 20° (*right*). The X- and Y-coordinates of the imaging plane are associated with spherical coordinates (*θ*, $\phi$) by Eq. (3).

A quasi-monochromatic gamma-ray beam produced by laser inverse Compton scattering was used in the experiment to verify the imaging performance of the prototype 3D-PSCC. Although there is a slight energy distribution due to the physical property of inverse Compton scattering^22^, the energy peaks of the gamma-ray beam are 1.7 MeV and 3.9 MeV, determined by the wavelength of the laser and the energy of electrons. We irradiated 17 positions of 3D-PSCC with the 1.7 MeV gamma-ray beam, and the center with the 3.9 MeV beam. Figure 1 shows images of the 1.7 MeV (*upper*) and 3.9 MeV (*lower*) gamma-ray beam with an incident angle of 0° (*left*) and 20° (*right*). The energy window was 1.4–1.9 MeV for 1.7 MeV imaging, and 3.0–4.2 MeV for 3.9 MeV. Both 1.7 MeV and 3.9 MeV images appeared concentrated around the position corresponding to each incident angle. Specifically, the peak locations derived from 1-D projection along the X-axis were 1.7° ± 1.1° and 19.6° ± 1.4° for each 1.7 MeV image, and 1.4° ± 2.4° and 17.4° ± 2.1° for each 3.9 MeV image. As the multi-pixel photon counter (MPPC) of the scatterer would be saturated above 1 MeV, the energy cut of the scatterer was set so as not to exceed 1 MeV. Therefore, only small Compton cones were permitted for 3.9 MeV, and as a result, only a narrow region within ±40° was captured in the 3.9 MeV image.

To evaluate the angular resolution of the 3D-PSCC, we used Angular Resolution Measure (ARM), which represents the difference between the geometrical scatter angle and the scatter angle calculated using energy deposit. The ARM spectra are displayed next to each image in Fig. 1. An experimental value of the ARM spectra was fitted by Gaussian to evaluate angular resolution. The angular resolution at 1.7 MeV was estimated as 3.4° ± 0.1° (FWHM) and 3.8° ± 0.1° (FWHM) for each incident angle. These experimental values and their dependence on the incident angles agreed well with Geant4 simulation. Note that the angular resolution of COMPTEL aboard CGRO was 3.9° (FWHM) at 1.7 MeV^5^. Moreover, the angular resolution at 3.9 MeV was 4.0° ± 0.5° and 4.6° ± 0.4° for each incident angle, which is again consistent with the simulation.

### Simultaneous drawing of gamma-ray sources from two directions

**Figure 2:**
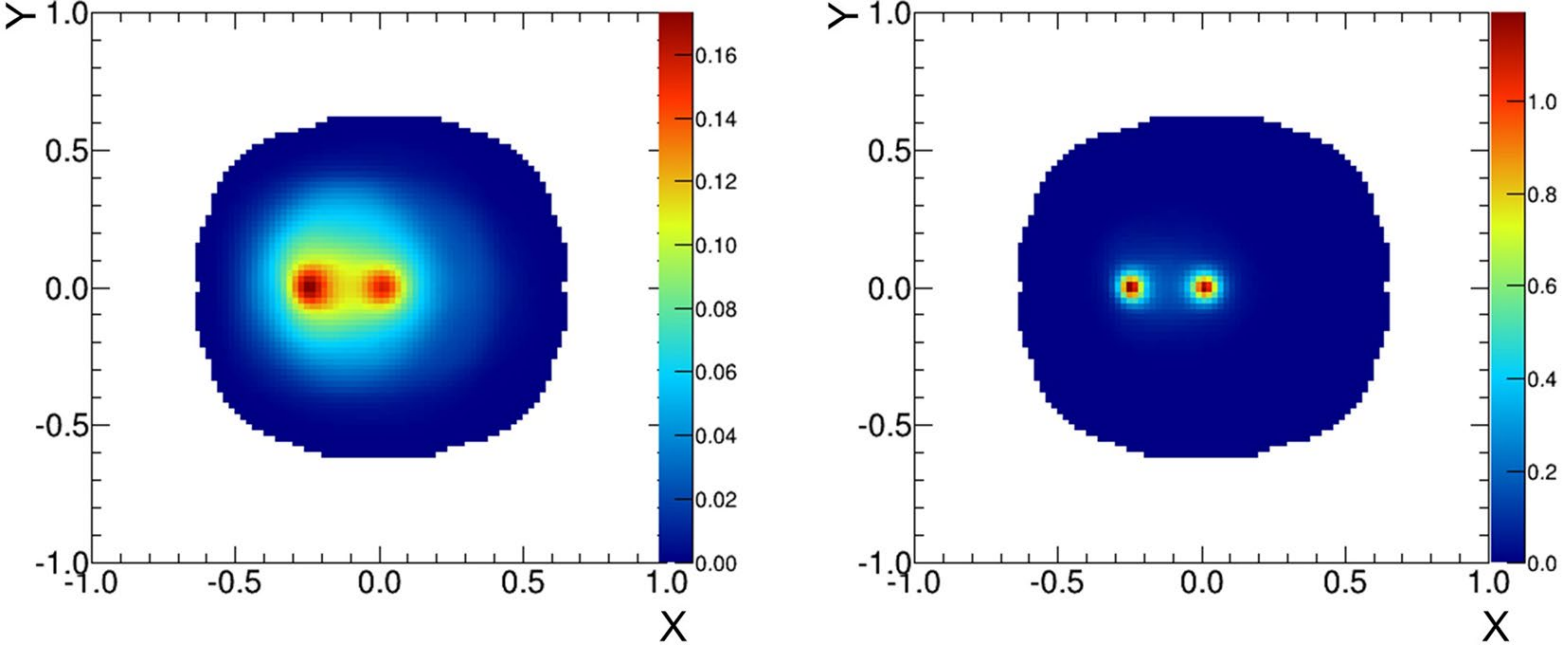
Images of 1.7 MeV gamma-ray beams irradiating from 0° and 20° off the axis of the camera. (*left*) Simple back projection image. (*right*) ML-EM image (iteration: 5 times).

In actual astronomical observation, we need to image gamma rays with different incident angles corresponding to different sources in the FoV. To mimic such a situation, we superposed an image of 1.7 MeV gamma-ray beams with incident angles of 0° and 20°. Flux and measurement time are the same for both 0° and 20°. The intensities of the two sources, however, will not be identical without correction because a Compton camera shows a different response to each source within the FoV. Therefore, we inspected the response of the 3D-PSCC to the sources which were distributed uniformly on a hemispheric surface using Geant4 simulation beforehand. The generated *sensitivity maps* were used to correct the intensity of gamma-ray sources within the FoV. Figure 2 (left) shows a simple back-projection image of two gamma-ray beams. The good angular resolution of the 3D-PSCC at 1.7 MeV enables the images of the two sources to be clearly separated. The maximum likelihood-expectation maximization (ML-EM) analysis was also performed, as shown in Fig. 2 (right). Further, the intensities of the two gamma-ray beams from 0° and 20° were nearly the same, as expected. This means the sensitivity map correctly anticipated the response of the 3D-PSCC to the sources at 0° and 20°.

## Discussion

**Figure 3:**
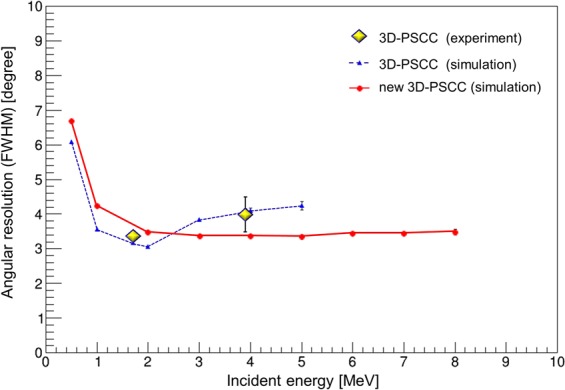
Comparison of the angular resolution simulated using Geant4 and the experimental value. Experimental value: *yellow plots*, simulation values of the prototype 3D-PSCC: *blue*, simulation values of the new 3D-PSCC: *red*.

The results of the experiment are validated by Geant4 simulation. Figure 3 shows the simulated angular resolution of the 3D-PSCC at various incident energies, compared with the experimental value. The energy window for the simulation was $0.9{E}_{i} < E < 1.1{E}_{i}$, where *E*_*i*_ denotes the incident energy of gamma rays. The angular resolution of a Compton camera is affected by energy resolution, position resolution and the Doppler broadening effect^23^. In this case, the energy resolution dominates the angular resolution below 1 MeV. However, position resolution, which is determined by the pixel size of the scatterer and absorber, dominates the angular resolution above 1 MeV. The Doppler broadening effect is caused by a momentum distribution of bound atomic electrons, but its influence on the angular resolution is negligible in the MeV-energy range.

The results in the experiment (*yellow plots*) agreed well with the Geant4 simulation (*blue*). Both the experiment and the simulation confirmed that the 3D-PSCC has better angular resolution than COMPTEL in the energy range below 2 MeV, despite its compact structure. However, the angular resolution of the 3D-PSCC decreases in the range above 3 MeV. In the high-energy range, energy uncertainty caused by the increased escape events dominates the angular resolution. As gamma rays above 3 MeV are considerably more energetic and cannot be easily stopped with the current configuration of 3D-PSCC, we applied a broad energy window to increase the number of proper events for imaging. The broad energy window also contains a substantial number of escape events where only a part of the incident energy was deposited within the detector. The discrepancy between the incident energy and the observed one caused degradation of the angular resolution, which led to the results in the energy range above 3 MeV.

As the prototype is not optimized for astronomical observation, the scintillators of the scatterer and absorber are not thick enough to obtain a sufficient number of whole-absorption events. To achieve high efficiency while maintaining high angular resolution in the MeV-energy range, a configuration of 3D-PSCC was briefly improved in simulation for future astronomical observation. Specifically, two layers of 10 mm thick scatterer and 60 mm thick 3-D position-sensitive absorber were placed 70 mm apart from each other. The detection area of the new 3D-PSCC assumed here was 20 × 20 cm^2^. Note that the new 3D-PSCC still has a compact structure compared to COMPTEL and can be loaded in 50 kg-class small satellites. High angular resolution was maintained in the energy range below 2 MeV with the new configuration (Fig. 3, *red*); 4.23° ± 0.02° at 1 MeV and 3.46° ± 0.03° at 2 MeV were achieved, respectively, which are still comparable to the corresponding values of COMPTEL. Furthermore, the angular resolution above 3 MeV was significantly improved owing to the reduction of escape events.

**Figure 4:**
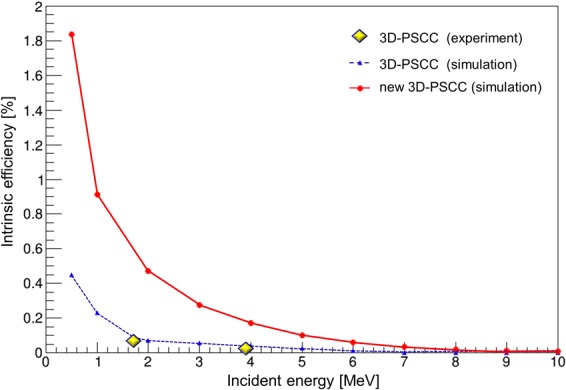
The simulated intrinsic efficiency of the 3D-PSCC. Experimental value: *yellow plots*, simulation values of the prototype 3D-PSCC: *blue*, the new 3D-PSCC: *red*.

The intrinsic efficiency of the 3D-PSCC was also estimated based on the simulation. In this simulation, the parallel gamma rays of each incident energy irradiated the camera uniformly at normal incidence. Figure 4 shows the estimated intrinsic efficiency for each incident energy. The experimental values (*yellow plots*) agreed well with the simulation (*blue*), but there is a slight discrepancy between the two. One factor that accounts for the discrepancy is that the gamma-ray background cannot be considered in the simulation due to the difficulty of estimating the background in the hutch. The intrinsic efficiency of the 3D-PSCC has been significantly improved between the prototype and the new configuration (*red*). The new 3D-PSCC shows an excellent performance, especially in the range below 2 MeV. In the high energy range, however, most gamma rays undergo multiple scattering events within the detector. The 3D-PSCC cannot recognize the sequence of interaction because the pixels are small and fairly close to each other. Therefore, the multiple scattering events are eliminated using a method described in *Methods*. On the other hand, the cells of COMPTEL are substantially larger (for example, the cell of the scatterer is 28 cm in diameter and 8.5 cm deep) and further apart from each other. Hence, the multiple scattering events within each cell are safely used for image reconstruction, and high efficiency is maintained in the case of COMPTEL.

We briefly discuss the anticipated performance of the new 3D-PSCC in terms of SΩ, where S is an effective area and Ω is the FoV at a corresponding energy. Note that a large SΩ is generally required for wide-field survey without focusing detectors such as the COMPTEL, Fermi-LAT and SPI/INTEGRAL. For example, SΩ of COMPTEL at 1 MeV is 15 cm^2^sr, and at 2 MeV is 20 cm^2^sr, respectively, assuming an FoV of 1 sr. Detector size of the new 3D-PSCC, 20 × 20 cm^2^, is more than an order of magnitude smaller than that of the COMPTEL, but the intrinsic efficiency is higher because the scatterer and absorber are placed much closer to each other. For the same reason, the new 3D-PSCC can capture an image of a gamma-ray source even with an inclination of 60°, thus achieving a wider FoV of ~3 sr. As a consequence, SΩ of the 3D-PSCC is estimated to be 11 cm^2^sr at 1 MeV and 6 cm^2^sr at 2 MeV, respectively. Although the difference between the SΩ of COMPTEL and 3D-PSCC is increased at higher energy, this first-order estimation suggests an interesting possibility that even a small 50-kg class satellite mission may be useful for future astronomical observations in the MeV energy window.

**Figure 5:**
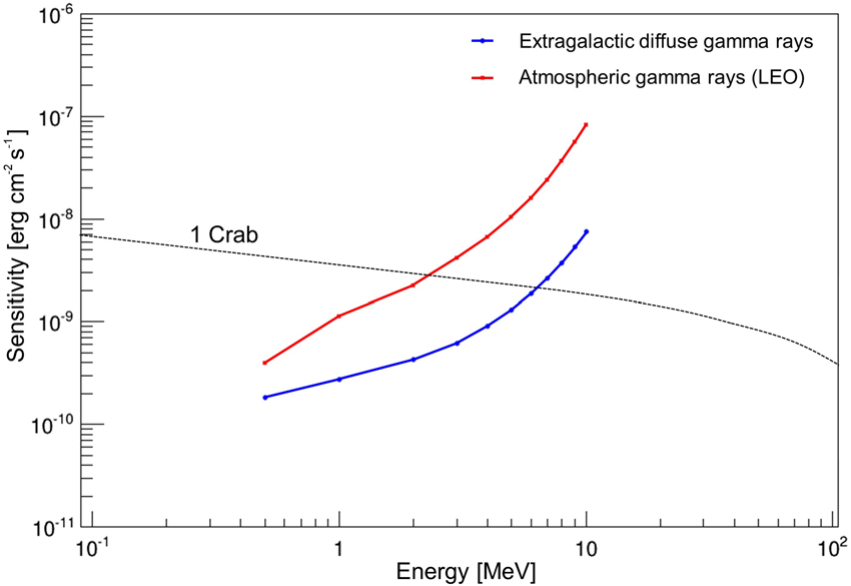
Provisional detection sensitivity of the new Compton camera ($3\sigma ,\,\Delta E=E,\,T=1\,{\rm{Msec}}$), considering the extragalactic diffuse gamma rays (Ackermann *et al*.^24^) and atmospheric gamma rays (De Angelis *et al*.^12^). The LEO was assumed to be the orbit of the small satellite.

Finally, we calculated the detection sensitivity of the new 3D-PSCC, taking into account the contamination of the extragalactic diffuse gamma rays^24^ and atmospheric gamma rays^12^. The low earth orbit (LEO) was assumed as the possible orbit of the small satellite. The calculated detection sensitivity for 1 Ms observation is shown in Fig. 5. The sensitivity for the atmospheric gamma ray is still preliminary because we have not yet employed background suppression tools such as the anticoincidence system for the new 3D-PSCC. However, this estimation suggests that the Crab Nebula, one of the brightest gamma-ray sources, can be detected with the new 3D-PSCC in the range below 2 MeV with a reasonable exposure time of 1 Ms.

We are aware that there are still number of problems we have to consider for actual astronomical use. Although we have developed a method to eliminate misdetection of signal gamma rays, atmospheric gamma rays or secondary particles in a satellite orbit can also cause background contamination. To estimate the effect of these background components, further analysis would be required, including a more elaborate design of the anticoincidence system and radiation tolerance of the detectors. Moreover, the materials that surround the 3D-PSCC like satellite housing, and even the detector itself, may also deteriorate the performance due to activation in the orbit. We have not taken these effects into account in the simulation yet. These topics would be addressed in future studies.

## Methods

### 3D-PSCC for imaging MeV gamma rays

**Figure 6:**
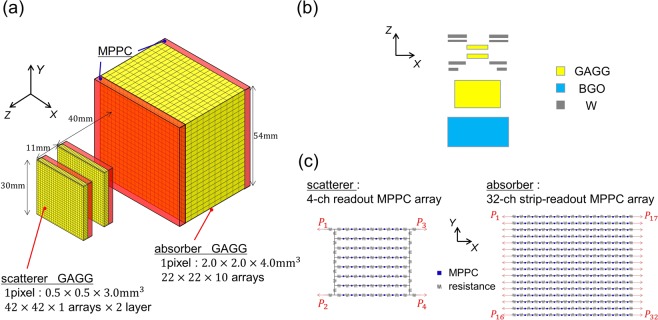
(**a**) Schematic view of the scatterer and absorber. Scatterers are surrounded by collars of tungsten passive shield to prevent direct injection to the absorber. (**b**) A configuration of the bismuth germanate (BGO) active shield and tungsten passive shield. (**c**) Schematic view of the MPPC arrays.

The 3D-PSCC was originally developed for imaging MeV gamma rays in proton therapy^21^. The target energy band of the 3D-PSCC is from 300 keV to 5 MeV. A schematic view of the 3D-PSCC is shown in Fig. 6. Two scatterers comprising 42 × 42 × 1 arrays of 0.5 × 0.5 × 3.0 mm^3^ cubes were placed apart from each other by 11 mm. In contrast, 22 × 22 × 10 arrays of 2.0 × 2.0 × 4.0 mm^3^ cubes were used as an absorber. Each pixel was divided by a reflector (BaSO_4_ with thickness of about 0.1 mm) in the XY direction defined in Fig. 6(a). The absorber’s pixels aligned along the Z direction were also divided by a thin layer of air, enabling easier measurement of the depth of interaction (DOI). Moreover, a bismuth germanate (BGO) active shield was placed behind the absorber, while tungsten passive shields were placed in front of the scatterer and absorber, as shown in Fig. 6(b).

Each scatterer was coupled with MPPC arrays (Hamamatsu Photonics S13361-3025NE-08) to acquire the information with respect to the deposited energy and its position. MPPC arrays were also placed at both the top and bottom of the absorber, to enable measurement of DOI^19^. To reduce the number of readout channels, each MPPC was divided by a resistor chain network, as shown in Fig. 6(c). The original position of signals can be identified by measuring the ratio of the amount of charge flowing along each side of a resistor chain. Although some pixels are degenerated near the edge of arrays due to light leakage, most of the pixels are clearly separated, and the typical spatial resolution is equal to the pixel size. We derived the energy spectra of 8,368 pixels individually, and then calibrated the gain of each pixel with various calibration sources before the experiment. The typical energy resolution of each pixel was 8.9% (FWHM) at 662 keV and 7.0% (FWHM) at 1.17 MeV. The dynamic range of each scatterer’s pixel was from ~10 keV to 1 MeV, while that of the absorber’s pixel was from ~50 keV to ~5 MeV. Four-channel readout MPPC arrays were coupled with the scatterer, while 32-channel strip-readout MPPC arrays were coupled with the absorber. Each interaction position of the scatterer was calculated using the following equation:

1 $$X=\frac{L\{({P}_{3}+{P}_{4})-({P}_{1}+{P}_{2})\}}{{P}_{1}+{P}_{2}+{P}_{3}+{P}_{4}},\,Y=\frac{L\{({P}_{1}+{P}_{3})-({P}_{2}+{P}_{4})\}}{{P}_{1}+{P}_{2}+{P}_{3}+{P}_{4}},$$

where *P*_*i*_ denotes the pulse height from the *i*th channel, and *L* denotes the half-length of the scintillator array in the XY direction. The X and Y coordinates of the absorber were calculated using the MPPC arrays on the top and bottom individually, while the Z-coordinate was identified using all 64 channels as in the following equation:

2 $$\begin{array}{rcl}{X}_{k} & = & \frac{W(\,-{\sum }_{i=1}^{16}\,{P}_{k,i}+{\sum }_{i=17}^{32}\,{P}_{k,i})}{{\sum }_{i=1}^{32}\,{P}_{k,i}},\\ {Y}_{k} & = & \frac{W\{\frac{15}{15}({P}_{k,1}+{P}_{k,17})+\frac{13}{15}({P}_{k,2}+{P}_{k,18})+\cdots +\frac{-15}{15}({P}_{k,16}+{P}_{k,32})\}}{{\sum }_{i=1}^{32}\,{P}_{k,i}},\\ Z & = & \frac{d\,{\sum }_{i=1}^{32}\,(\,-\,{P}_{1,i}+{P}_{2,i})}{{\sum }_{i=1}^{32}\,({P}_{1,i}+{P}_{2,i})}\end{array}$$

where $k=1,2$ denotes each MPPC array on the top and bottom of the absorber, *P*_*k*,*i*_ denotes the pulse height from the *i*th channel, *W* denotes the half-length of the scintillator array in the XY direction, and *d* denotes the half-length of the scintillator array in the Z direction.

The signals from each MPPC array were sent to a signal-processing board via FPC cables. Subsequently, the time-stamped ADC data were translated to binary data in FPGA, which were sent to a laptop via USB 3.0 cable. The output data were recorded in list mode, and then time coincidence analysis was performed offline. The coincidence time window was 1.5 *μ*s in this experiment. The operation voltage of each MPPC array was controlled by applying 5.0 V to the signal-processing board.

Table 1 shows the energy cut range that is applied for each incident energy and angle. In particular, the *E*_1_ cut range was determined so that the scattering angle would be below 20°, which is approximately the maximum angle allowed for the configuration of the current detector. With this energy cut, we can remove the back-scattering events in which the incident gamma ray first hits the absorber and is then subsequently absorbed in the scatterer. We also did not use events with *E*_1_ > 1000 keV because the MPPC of the scatterer may be affected by the saturation of scintillation light (see Results section). Furthermore, the *E*_1_ cuts of the simulation were set in the same manner as the experiment.

**Table 1:** The energy cut range applied for MeV gamma-ray imaging.

Incident energy [keV]	Incident angle [degree]	*E*_1_ (scatterer) and *E*_2_ (absorber) [keV]	*E*_1_ (scatterer) [keV]
1700	0	1400 < *E*_1_ + *E*_2_ < 1900	10 < *E*_1_ < 200
1700	20	1400 < *E*_1_ + *E*_2_ < 1900	10 < *E*_1_ < 200
3900	0	3000 < *E*_1_ + *E*_2_ < 4200	10 < *E*_1_ < 230; 500 < *E*_1_ < 1000
3900	20	3000 < *E*_1_ + *E*_2_ < 4200	100 < *E*_1_ < 200; 450 < *E*_1_ < 1000

**Figure 7:**
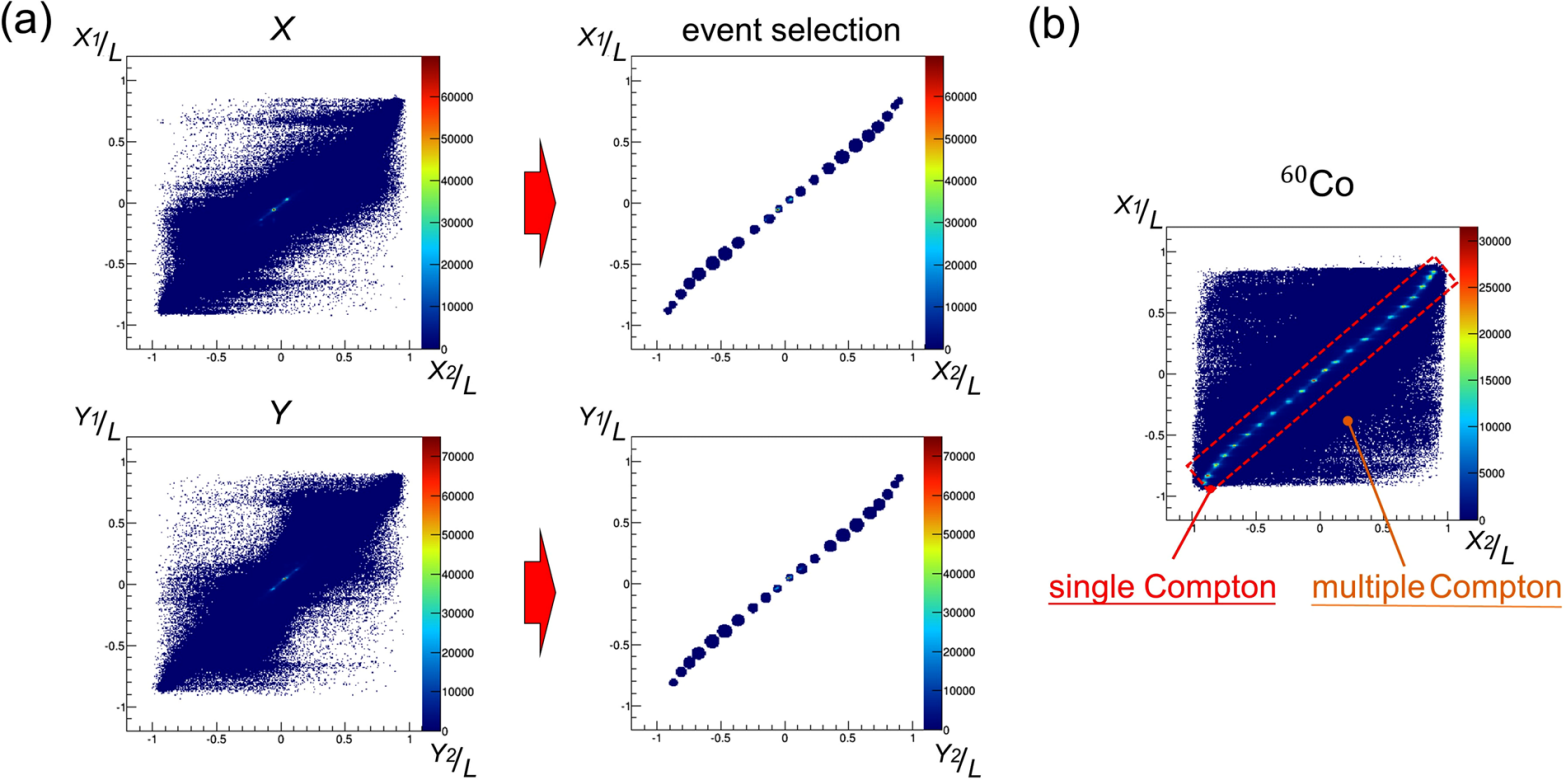
(**a**) 2-D histograms comparing *X*_1_/*L* vs *X*_2_/*L* and *Y*_1_/*L* vs *Y*_2_/*L* in the absorber, where *X*_*k*_ and *Y*_*k*_ denote the coordinates of each MPPC array, and *L* denotes the half-length of the absorber. (**b**) 2-D histogram comparing *X*_1_/*L* vs *X*_2_/*L* using a ^60^Co source. Each peak in the red box represents a single Compton event, while multiple Compton events appear outside the red box. The event selection range in (**a**) was determined using a ^60^Co source.

The major types of background in MeV gamma-ray imaging are escape, back-scattering and multiple scattering events. As each pixel of the 3D-PSCC is small and they are placed side by side, the sequence of multiple scattering within a detector cannot be recognized. Therefore, we have to eliminate multiple scattering events to improve the signal-to-noise ratio. To reject these events, we compared interaction points calculated individually from each MPPC array of the absorber using Eq. (2). If the photon undergoes single Compton scattering, the interaction points calculated from the top and bottom of the absorber should be identical to each other. Figure 7(a) shows a 2-D plot comparing the coordinates of the top and bottom in the case where a 1.7 MeV beam irradiated the center of the 3D-PSCC, as an example. We pre-emptively determined the event selection range using a ^60^Co source (shown in Fig. 7(b)). By applying this method, 71% and 79% of all the coincidence events were rejected as multiple scattering events for 1.7 MeV and 3.9 MeV, respectively. In addition, both escape and back-scattering events are rejected by the energy cuts. As the scatterer and absorber are close to each other, the 3D-PSCC cannot distinguish between the proper events and back-scattering events. While COMPTEL measures ToF to eliminate the back-scattering events, the 3D-PSCC used the energy cut of the scatterer to avoid back-scattering gamma rays from the absorber. Escape events were also excluded by veto signals from the BGO active shield.

In the process of image reconstruction, the directions of sources are calculated in spherical coordinates (*θ*, $\phi$). Therefore, each pixel of the imaging plane (*R*, $\Theta$) has to correspond to that of the sphere on a one-to-one basis. We adopted equisolid angular projection represented by the following equation:

3 $$R=\sqrt{2}\,\sin \,\frac{\theta }{2},\,\Theta =\phi \,(X=R\,\cos \,\Theta ,\,Y=R\,\sin \,\Theta )$$

### Description of the experiment in NewSUBARU

NewSUBARU is a synchrotron radiation facility located in the site of SPring-8, Hyogo in Japan. High-energy electrons from Spring8 are used for various experiments in 11 beam lines. Our experiment was conducted in BL01, which treats a quasi-monochromatic MeV gamma-ray beam. The gamma rays are produced by inverse Compton scattering of a CO_2_ laser (*λ* = 10.6 *μ*m) with 1 or 1.5 GeV electrons. Here, the upper limits of incident energy were estimated at 1.7 MeV and 3.9 MeV. These gamma rays pass through a thick Pb collimator to form a quasi-monochromatic gamma-ray beam. The experimental conditions of the gamma-ray beams are shown in Table 2.

**Table 2:** Experimental conditions for MeV gamma-ray imaging.

gamma-ray energy [MeV]	laser wavelength [mm]	laser power [W]	electron energy [GeV]	electron beam current [mA]	collimator [mm]
1.7	10.6	3.3	1	300	3
3.9	10.6	1.5	1.5	222	4

**Figure 8:**
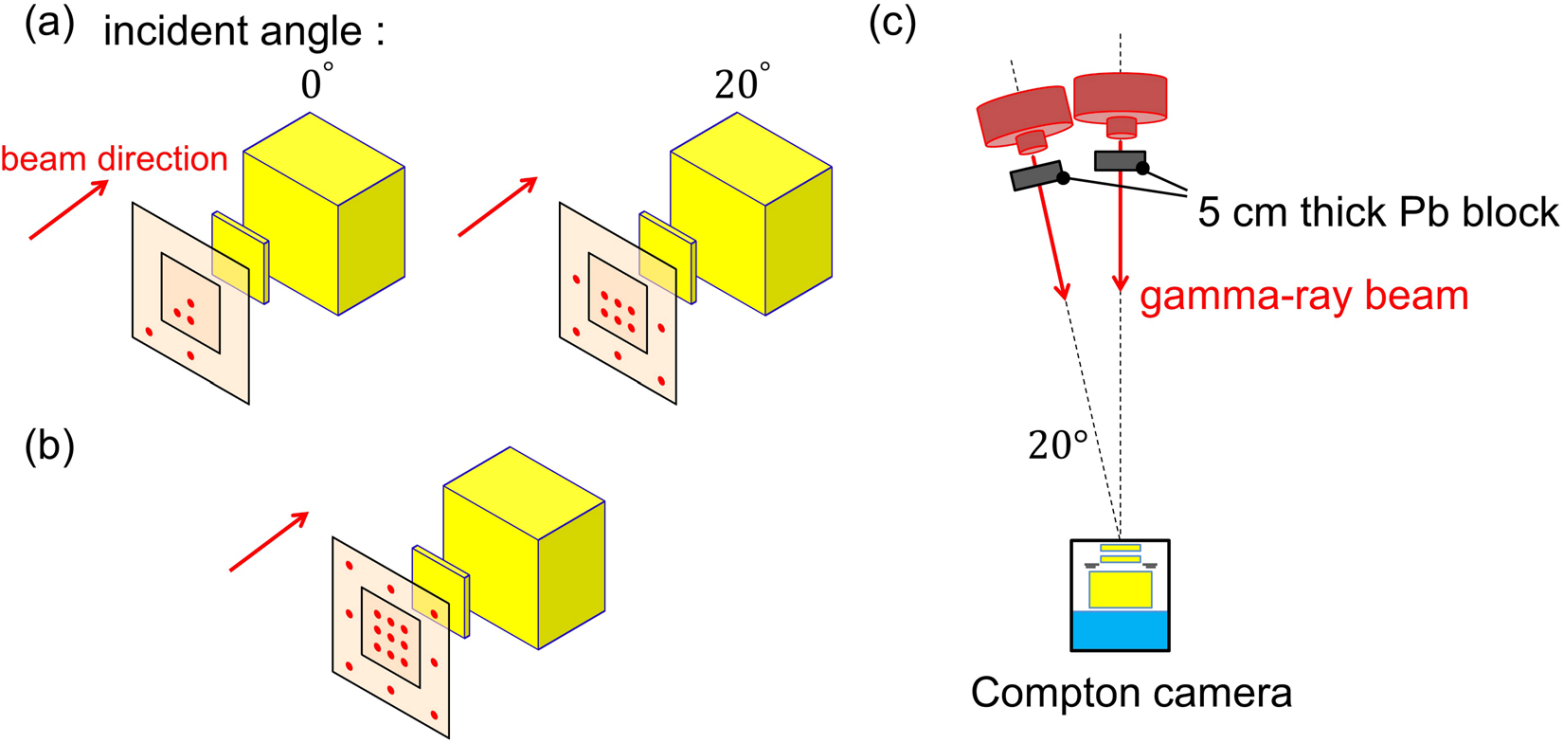
(**a**) Positions irradiated with a 1.7 MeV gamma-ray beam for each incident angle, represented as red points. (**b**) All 17 positions covered considering the symmetry of the camera. (**c**) Experimental setup for imaging MeV gamma-ray beams.

To simulate the situation of an astronomical observation, we used the MeV gamma-ray beam assuming signals from celestial gamma-ray sources. Considering the symmetry of the camera, irradiating points of 1.7 MeV beams (shown in Fig. 8(a)) are selected to cover 17 positions (shown in Fig. 8(b)) so that the 3D-PSCC is exposed to gamma rays as uniformly as possible. Eight out of these 17 positions are assuming background events that enter the absorber directly. By contrast, only the center of the 3D-PSCC was irradiated with the 3.9 MeV gamma-ray beam due to time restrictions of the experiment. A geometry of the experiment is shown in Fig. 8(c). A 5 cm thick Pb block was placed in front of an irradiating port considering the rate tolerance of the 3D-PSCC. The measurement times for 1.7 MeV and 3.9 MeV were 5 min and 30 min respectively.

## References

[1] Ubertini P (2003). IBIS: The Imager on-board INTEGRAL. Astronomy & Astrophysics.

[2] Bird AJ (2010). The Fourth IBIS/ISGRI Soft Gamma-ray Survey Catalog. The Astrophysical Journal Supplement Series.

[3] Atwood WB (2009). The Large Area Telescope on the Fermi Gamma-Ray Space Telescope Mission. The Astrophysical Journal.

[4] Acero F (2015). Fermi Large Area Telescope Third Source Catalog. The Astrophysical Journal Supplement Series.

[5] Schönfelder V (1993). Instrument description and performance of the Imaging Gamma-Ray Telescope COMPTEL aboard the Compton Gamma-Ray Observatory. The Astrophysical Journal Supplement Series.

[6] Schönfelder V (2000). The first COMPTEL source catalogue. Astron. Astrophys. Suppl. Ser..

[7] Diehl R (1995). Comptel observations of Galactic ^26^Al emission. Astronomy & Astrophysics.

[8] Bouchet L, Jourdain E, Roques J-P (2015). The Galactic ^26^Al Emission Map as Revealed by INTEGRAL SPI. The Astrophysical Journal.

[9] Iyudin AF (1994). COMPTEL observations of Ti-44 gamma-ray line emission from Cas A. Astronomy & Astrophysics.

[10] Kozlovsky B, Murphy RJ, Ramaty R (2002). Nuclear deexcitation gamma-ray lines from accelerated particle interactions. The Astrophysical Journal Supplement Series.

[11] Dogiel V (2009). Nuclear Interaction Gamma-Ray Lines from the Galactic Center Region. Astronomy and Astrophysics.

[12] De Angelis A (2017). The e-ASTROGAM mission Exploring the extreme Universe with gamma rays in the MeV – GeV range. Experimental Astronomy.

[13] Tanimori T (2017). Establishment of Imaging Spectroscopy of Nuclear Gamma-Rays based on Geometrical Optics. Scientific Reports.

[14] Watanabe S (2014). The Si/CdTe semiconductor Compton camera of the ASTRO-H Soft Gamma-ray Detector (SGD). Nuclear Instruments and Methods in Physics Research Section A: Accelerators, Spectrometers, Detectors and Associated Equipment.

[15] Bandstra MS (2011). Detection and Imaging of the Crab Nebula with the Nuclear Compton Telescope. The Astrophysical Journal.

[16] Kataoka J (2013). Handy Compton camera using 3D position-sensitive scintillators coupled with large-area monolithic MPPC arrays. Nuclear Instruments and Methods in Physics Research Section A: Accelerators, Spectrometers, Detectors and Associated Equipment.

[17] Kishimoto A (2017). Development of a compact scintillator-based high-resolution Compton camera for molecular imaging. Nuclear Instruments and Methods in Physics Research Section A: Accelerators, Spectrometers, Detectors and Associated Equipment.

[18] Yoneyama M (2018). Evaluation of GAGG:ce scintillators for future space applications. Journal of Instrumentation.

[19] Kishimoto A (2013). Development of a Dual-Sided Readout DOI-PET Module Using Large-Area Monolithic MPPC-Arrays. IEEE Transactions on Nuclear Science.

[20] Koide A (2018). Precision imaging of 4.4 MeV gamma rays using a 3-D position sensitive Compton camera. Scientific Reports.

[21] Mochizuki S (2019). High-precision compton imaging of 4.4 MeV prompt gamma-ray toward an on-line monitor for proton therapy. Nuclear Instruments and Methods in Physics Research Section A: Accelerators, Spectrometers, Detectors and Associated Equipment.

[22] Amano S (2009). Several-MeV *γ*-ray generation at NewSUBARU by laser Compton backscattering. Nuclear Instruments and Methods in Physics Research Section A: Accelerators, Spectrometers, Detectors and Associated Equipment.

[23] Zoglauer A, Kanbach G (2003). Doppler broadening as a lower limit to the angular resolution of next generation Compton telescopes. Proceedings of SPIE.

[24] Ackermann M (2015). The Spectrum of Isotropic Diffuse Gamma-Ray Emission between 100 MeV and 820 GeV. The Astrophysical Journal.

